# Non-Thermal Effects of Terahertz Radiation on Gene Expression: Systematic Review and Meta-Analysis

**DOI:** 10.3390/genes15081045

**Published:** 2024-08-08

**Authors:** Mactar Ndiaga Dione, Sen Shang, Qi Zhang, Sicheng Zhao, Xiaoyun Lu

**Affiliations:** 1 School of Life Science and Technology, Xi’an Jiaotong University (XJTU), Xi’an 710049, China; 2 Key Laboratory of Biomedical Information Engineering of Ministry of Education, School of Life Science and Technology, Xi’an Jiaotong University, Xi’an 710049, China

**Keywords:** terahertz radiation, gene expression profile, pathway analysis

## Abstract

With the advancement of terahertz technology, unveiling the mysteries of terahertz has had a profound impact on the field of biomedicine. However, the lack of systematic comparisons for gene expression signatures may diminish the effectiveness and efficiency of identifying common mechanisms underlying terahertz effects across diverse research findings. We performed a comprehensive review and meta-analysis to compile patterns of gene expression profiles associated with THz radiation. Thorough bibliographic reviews were conducted, utilizing the PubMed, Embase, Web of Science, and ProQuest databases to extract references from published articles. Raw CEL files were obtained from Gene Expression Omnibus and preprocessed using Bioconductor packages. This systematic review (Registration No. CDR42024502937) resulted in a detailed analysis of 13 studies (14 papers). There are several possible mechanisms and pathways through which THz radiation could cause biological changes. While the established gene expression results are largely associated with immune response and inflammatory markers, other genes demonstrated transcriptional outcomes that may unravel unknown functions. The enrichment of genes primarily found networks associated with broader stress responses. Altogether, the findings showed that THz can induce a distinct transcriptomic profile that is not associated with a microthermal cellular response. However, it is impossible to pinpoint a single gene or family of genes that would accurately and reliably justify the patterns of gene expression response under THz exposure.

## 1. Introduction

Terahertz electromagnetic waves are a gap bridging the electronics and the photonics regions of the electromagnetic spectrum [1,2]. In the last two decades, researchers have realized that terahertz (THz) technology can be used for real-life applications. By definition, THz radiation has photon energy in the milli-electron volt (meV). Hence, it is physically impossible for THz radiation to ionize components of biomacromolecules such as DNA and proteins. The vibration and rotation energy levels of many biological macromolecules such as DNA, RNA, and lipids are in the THz band [3,4]. Frequencies from 0.10 to 2.53 THz are the most investigated in the published studies. This could be explained by the fact that sources and detectors are more accommodating in these ranges [5]. Frequency becomes less penetrative as its value increases. In addition, a peak absorption is observed with biological tissues at 0.1 THz due to interactions between THz waves and water molecules increasing transcriptional activity [6].

The history of THz bioeffects studies has witnessed disinterest, rebirth, and promise. For a long time, this field has remained underdeveloped and inadequately explored because of a scarcity of appropriate sources and detection methods [7,8]. The advancement of manufacturing and the increased accessibility of THz-related instruments have propelled research on THz bioeffects and the field is now gaining momentum. One crucial aspect that was initiated early on and holds significant importance is the impact of terahertz radiation on gene expression.

More than 150 articles have been published on THz medical applications on PubMed, yet only around 50 address the effects on living cells and tissue [9]. The environmental exposures to THz have increased in the last two decades, and this trend may continue in the coming years.

Traditionally, the effects following the radiation of the magnitude of THz on biological molecules and the understanding of their mechanisms have not been of real concern [10,11]. On the one hand, the results of investigations are still pending as to whether exposure to THz should be prevented to avoid biological and potential health risks associated with the radiation energy emitted by widespread devices [11]. On the other hand, the question whether they should be reduced by limiting the deployment of these technologies until adequate safety standards or margins for longer periods are reached by consensus is still being debated [12]. Therefore, answers to queries related to the biological effects of terahertz radiation exposure on organisms are worth exploring.

The choice of the range of THz electromagnetic frequency is supported by the idea that this frequency increases water molecule rotation and interacts with biomolecules such as proteins. However, those claims are difficult to verify experimentally. Output power is the amount of energy emitted and absorbed in the biological material, leading to an increase in the thermal effects. The thermal effect following THz pulses absorption is, to an extent, connected to power density [13,14]. Computational and experimental models both showed the damage threshold to be 7.16 W/cm^2^. Uncontrolled output power and excessive thermal exposure cause structural changes and harmful thermal effects [15].

Alteration in gene expression is one of the first steps towards changes in intracellular signaling pathways and directly modifies cell dynamics and plasticity. It is argued that transcription activity regulators and macromolecular changes can be triggered by THz and lead to gene expression shifts. THz irradiation action on the latter is thought to be catalytic and linked to several interdependent factors such as transcription factors, promoters, and the pathways involved [16].

While non-ionizing, exposure to THz frequencies may hinder oxidative stress repair mechanisms, increasing the risk of cancer through the impairment of cellular components, including DNA [17]. In the present literature, reports on the efficacy of THz radiation as a biologic modulator exhibit inconsistency on various biological levels—some studies reported strong effects to subtle differences at the DNA level and some correlations between THz and biological effects [18,19,20], where a few studies documented small changes in the differential response of genes; some suggest its promising therapeutic potential because of its ability to boost immune cells [21,22]. However, the mechanisms underlying these changes remain elusive and the data used in the models related to the subject are very preliminary.

The systematization and standardization of the results is mainly impeded by fundamental differences in the experiment protocols.

The objectives of this review were as follows: (1) conduct an updated systematic review of the available literature on the relationships between terahertz radiation exposure and gene expression; (2) summarize key parameters in the experimental studies of terahertz irradiation; (3) highlight groups of genes and the pathways that are enriched. The outcome of this review could contribute to broadening the evidence of the terahertz radiation’s effect on gene expression and downstream consequences.

## 2. Materials and Methods

### 2.1. Protocol and Registration

The protocol of this research is available in PROSPERO (CDR42024502937). This systematic review adhered to the international standard called Preferred Reporting Items for Systematic Meta-Analyses (PRISMA) [23].

### 2.2. Eligibility Criteria

We included original articles up until 28 February 2024, including animals, cell, and in vitro models—the studies involved investigated terahertz irradiation followed by a gene expression profile assessment between irradiated and control groups. The reporting gene expression studies must have used high-throughput RNA sequencing or a microarray for quantification. The pre-specified exclusion criteria were as follows: (1) no relevant outcome measure reported; (2) gene expression measured using qPCR, (3) not an original full research paper (reviews papers, conference proceedings, books, editorials, comments, research letters, no detailed methodology described, as well as manuscripts without an English full text available). Finally, only articles published in English were included.

### 2.3. Information Sources

Electronic database (PubMed, Embase, Web of Science, and ProQuest) searches were conducted between 2 January 2024 and 1 March 2024.

### 2.4. Search

The full research strategies used within these databases are presented in Supplementary File S1 of this manuscript. The following components were included in the search string or other subject heading terms. The search string was conducted by M.N.D. The desired references were obtained by combining the main keywords and their synonyms. For example: (1) “terahertz radiation”, “terahertz wave”, “terahertz pulse”, and (2) “gene expression”, “gene expression profiling”, and “transcriptome” were some of the compound keywords.

### 2.5. Study Selection

Search results across databases were merged using Endnote. After duplicates were removed, two researchers (M.N.D, S.S) separately screened titles and abstracts of retrieved records for full-text selection. In the case of a disagreement on which article’s full text should be screened, consensus was reached by discussion. The full-text screening constituted the last step of the selection process. Thereafter, in pairs, (M.N.D, S.S) we independently screened full-text articles for inclusion. Again, in the case of a disagreement or uncertainties, consensus was reached based on inclusion and exclusion criteria or by discussion. A third party was consulted if necessary.

### 2.6. Data Collection Process

In this step, reviewers independently recorded relevant information from studies. A customized Excel sheet was used to extract the following: (1) author name; (2) year of publication; (3) country; (4) methodological information such as (a) animals/cell type models, (b) frequency range, (c) average power, (d) peak power, (e) pulse rate, repetition rate; (5) duration of exposure; (6) time before RNA extraction; (7) method of genetic analysis; (8) fold change; (9) number of differentially expressed genes; (10) pathways analysis method; and (11) biological outcomes. The GEO [24] search was conducted using (THz) OR (Terahertz). The results were further filtered using Entry type = Series, then Organism = mus musculus, and finally Study type = Expression profiling by array. Raw CEL files were downloaded using the GEOquery package (version 2.66.0) [25]. The full search string used was as follows: (Terahertz[All Fields] OR THz[All Fields]) AND “Mus musculus”[porgn] AND (“gse”[Filter] AND “Expression profiling by array”[Filter]).

### 2.7. Bioinformatical Analysis

All the following steps were performed in R (version 4.2.3), from Bioconductor (version 3.16) [26]. The raw expression files of the Affymetrix and Agilent files were normalized using the *fRMA* function in the *fRMA* package (version 1.50.0) [27] and quantile normalization, respectively, in R. Probe IDs were annotated to match the corresponding gene symbol. The limma R (version 3.54.2) package [28] was used to screen for DEGs separately between the control and the exposed group. DEGs were selected to have a logFC (log fold change) > 1 and a *p*-value < 0.05. Over-representation was performed with clusterProfiler (version 4.6.2) [29] using C5 canonical pathways from MsigDB (version 7.5.1) [30]. This was considered significative if the adjusted *p*.value was less than 0.05. The R-script from pre-processing to DEGs screening and enrichment analysis can be found in Supplementary File S1.

## 3. Results

### 3.1. Study Selection

**Figure 1:**
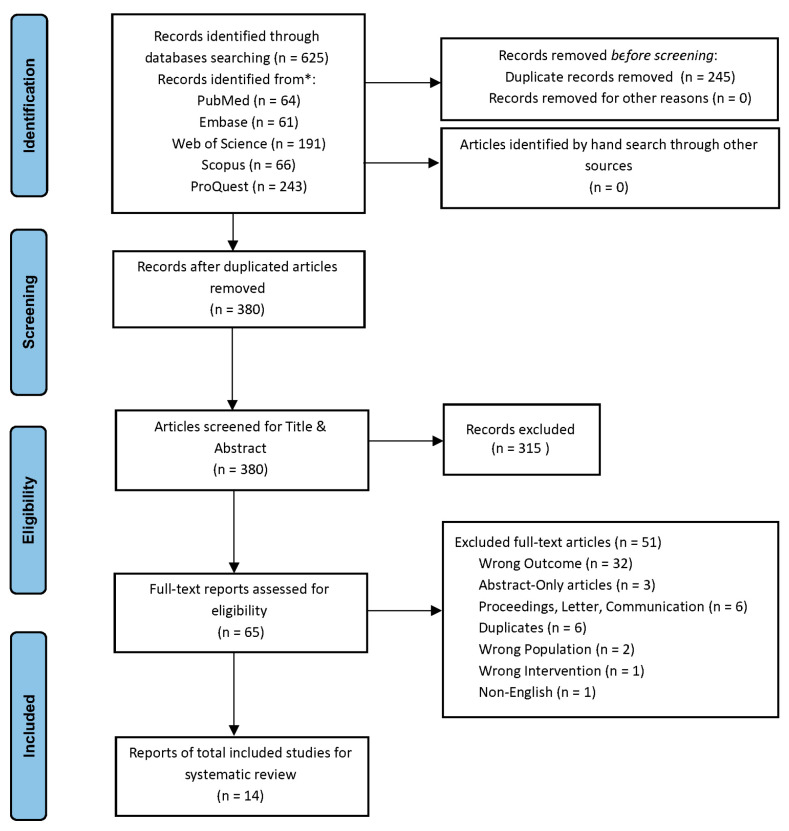
Study selection following Prisma flowchart.

After duplicated articles were removed, a total of 625 articles were retrieved, of which 64 were from PubMed, 61 from Embase, 66 from Scopus, 191 from Web of Science Core Collection, and 243 from ProQuest. Three-hundred and eighty (380) studies were found to be eligible for the review. After titles, the abstracts and keywords were analyzed; 65 articles were sought for full-text screening. When the final eligibility was manually screened, 14 articles were found to meet the inclusion criteria. Figure 1 shows the Prisma flowchart describing the process of the systematic literature search to identify all eligible studies. Thereafter, each of these papers were thoroughly and autonomously read by one of the reviewers to identify and extract meaningful information. The datasets comprising five microarrays were the following: SuperSeries [GSE41106] (GSE41083, GSE41084, GSE41085), GSE44671, and GSE178729; these were extracted from the Gene Expression Omnibus (GEO) using the GEOquery package [25].

### 3.2. Study Characteristics

The studies considered and their respective parameters are summarized in Table 1.

The countries that are a part of the affiliations in the studies are China (four articles), Canada (three articles), USA (three articles), Russia (two articles), and South Korea (two articles).

The analysis of the selected primary studies showed that they were conducted on eight different cellular models and one bacterial species (*E. coli*); the in vitro studies corresponded to C57BL/6J and BALB/c mice. It is essential to provide as many details as possible in any exposure study about the source of irradiation. We have listed the studies and summarized all the reported parameters in the studies.

As seen in Table 1, the parameters of the THz fluctuations in the irradiation sources in the experimental settings exhibit a manifest variability in their characteristics: frequency, intensity (average and peak power density), and operation regime (pulsed and CW). All experiments were carried out under monitored thermal conditions to ensure that temperature was not related to the observed effects.

The frequency of radiation in our studies varied from 0.1 THz [31] to 10 THz [10]. Exposure time varied substantially between 10 and 12 h. The shortest time exposure was 10 min [32] and the longest was 12 h exposure [16]. In most of the studies, the biological materials were harvested at one selected time point for mRNA quantification, from 10 min [33] to 48 h [34]. In one study, mRNA was extracted 28 days after the repeated treatment of THz exposure [22].

**Table 1:** Study characteristics of the included studies.

Study	Country	Biological Model	Sample (*n*)	THz Exposure Parameters								RNA Extraction Time Post-Exposure
				f (THz)	Pulse Rate	Pulse Duration	I Average (mW/cm^2^)	I Peak (mW/cm^2^)	T (°C)	Δ (°C) Increase	Exposure Duration	
In vitro studies												
Bock et al. [10]	USA	Mouse stem cells	Duplicates	~10	1 kHz	35 fs	1	~30	21.61	NC (0.28)	9 h	NA
Alexandrov et al. [16]	USA	Mouse stem cells	Triplicates	2.52	1 kHz	35 fs	~0	2	26–27	NC	12 h	Immediat.
Tivota et al. [32]	Canada	Artificial human skin tissue	Quadruplicates	0.2–2.5	1 kHz	NA	57	33	21	<0.7	10 min	30 min
Bogomazova et al. [35]	Russia	Human embryonic stem cells	Duplicates	2.3	CW	NA	110	NA	24	~1	1 h	2 h
Echchgadda et al. [36]	USA	Human jurkat T lymphocytes cell line	Triplicates	2.52	CW	NA	532	NA	37	~6	40 min	4 h
Hough et al. [37,38]	Canada	3D human skin tissue	Quadruplicates	0.6	>1 kHz	50 fs	74	73.8	37	<1	10 min	30 min
Zhao et al. [39]	China	RPE cells, HCE cells, Müller cells	Quadruplicates	0.7	1 kHz	0.5 ps	~1	2	~37	<0.2	6 h	15 h
Shang et al. [31]	China	Rat primary hippocampal neurons	Triplicates	0.1	NA	NA	33	NA	37	NC	20 min	2 h
Peltek et al. [33]	Russia	*E. coli*	Duplicates	1.25–3.75	5.6–22.4	40–100 ps	0.14	0.8	37	2	15 min	10 min
Zhao et al. [40]	China	Mouse cortical neurons and oligodendrocytes precursor cells	Quadruplicates	3.1	1 kHz	NA	70 μW/cm^2^	NA	35	NC	15 mn (x3)/day–3 h/day	NA
Cheon et al. [34]	South Korea	Melanoma cells	Triplicates	1.6	1 kHz	35 fs	750	NA	38	NA	30 min	0–48 h
Animal model studies												
Kim et al. [8]	South Korea	C57BL/6J and BALB/c	Triplicates	2.50	1 kHz	310	0.32 μW/cm^2^	NA	22	NC	1 h	24 h
Xu et al. [22]	China	Female C57BL/6	5–5	0.14	NA	NA	90	NA	RT	NC	10 min	28 days

Notes. f = frequency; I = power density (mW/cm^2^); I average = average incident irradiance; I peak = peak power density; T = ambient temperature; Immediat. = immediately; h = hour; min= minutes; Δ = increase on temperature during experiment; NC = no temperature change; NA = not reported; RT = room temperature.

All studies evaluated gene expression changes but differed widely in technologies for estimating gene expression changes and statistical cut-offs. All included studies measured gene expression adequately in accordance with the standard and indicated that the specific changes noticed may primarily depend on the general responsiveness of the biological material towards THz irradiation. Microarray was revealed to be the most popular method of investigating THz bioeffect.

Nine out of thirteen studies used gene expression microarrays to assess gene expression while four studies employed next-generation RNA-sequencing techniques with the Illumina platform. All studies resulted in two distinct expression patterns consisting of upregulation and downregulation. The gene expression technology used differentially expressed genes and databases for enrichment analysis and pathway analysis results; the biological outcomes for each study reported in the reviewed studies are synthesized in Table 2.

This search in GEO outputted seven results. After manual screening, only five studies were eligible (Table 3). One was a super-series of the three studies already available (GSE41106), and the other was composed of only one sample (GSE23888); therefore, these were not considered.

**Figure 2:**
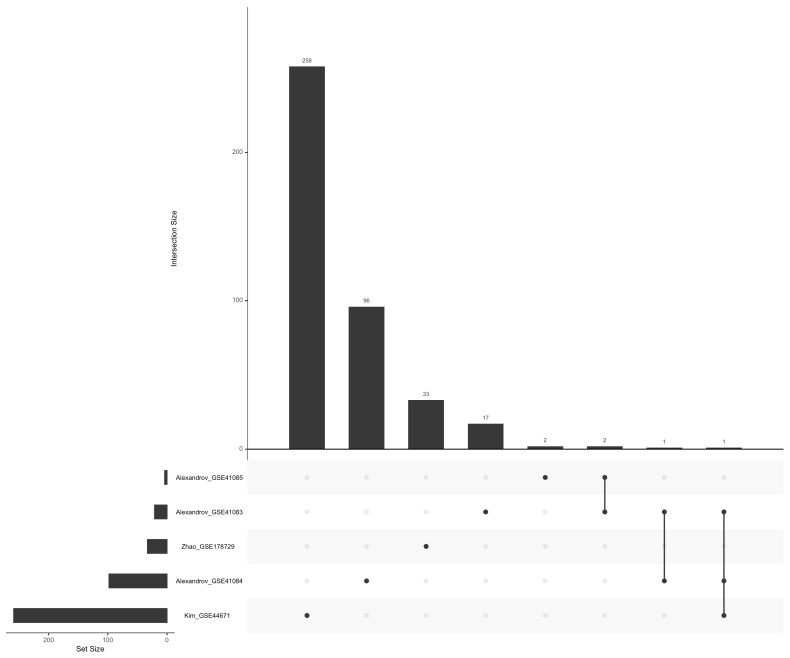
UpSet plot visualizing the distribution and overlap between sets of unique or shared differentially expressed genes and their intersection among microarray datasets (Alexandrov et al. [16]; Kim et al. [8]; Zhao et al. [40]). The horizontal bars depict the number of DEGs. The vertical bars display common elements in the sets, represented with dots under each bar.

**Figure 3:**
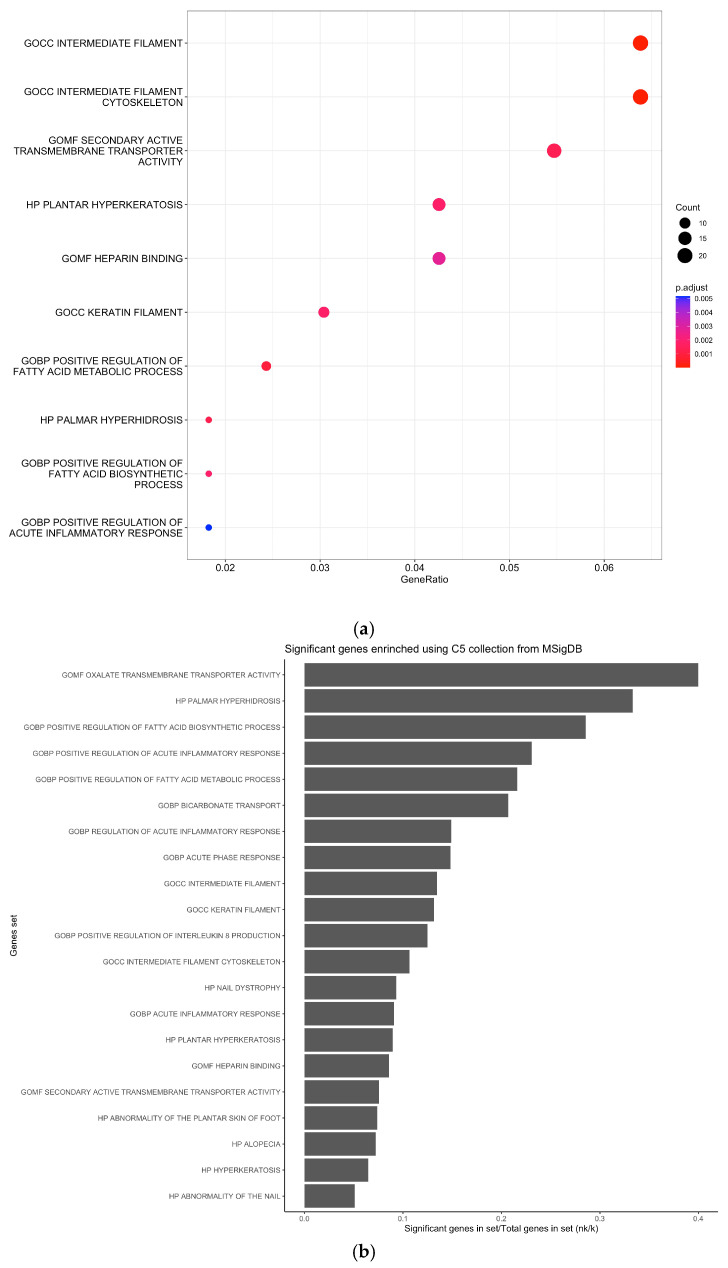

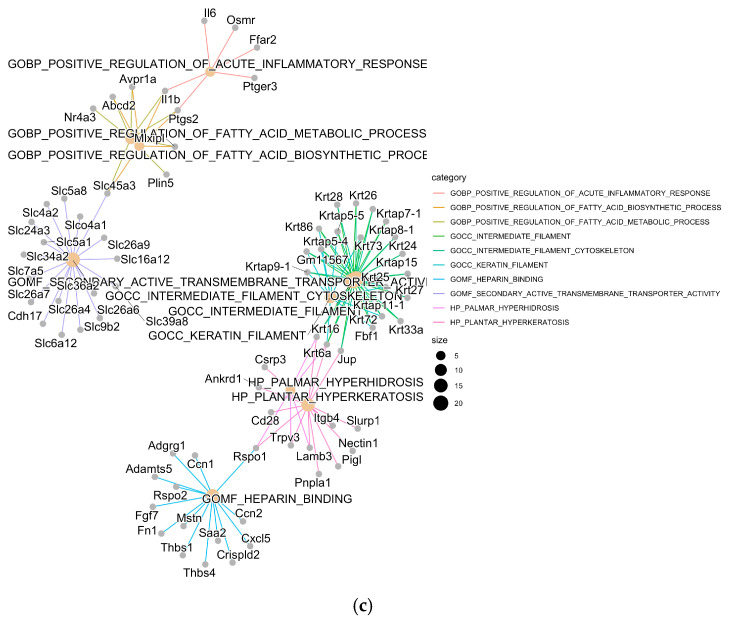
Enrichment analysis: (**a**) significant genes enriched from MsigDB (c5 canonical pathways); (**b**) significant genes in set/total genes in set (nk/k); (**c**) Cnet plot of significant genes enriched.

We performed a functional enrichment of the selected differentially expressed genes. The unique and shared DEGs are presented in an upset plot (Figure 2). We then constructed a network of the significantly enriched pathway from the five datasets retrieved in GEO, using Limma and ClusterProfiler. The results are presented in Figure 3. Among pathways that were the most enriched were “Oxalate Membrane Transporter Activity”, “Palmar Hyperhidrosis”, “Positive Regulation of Fatty Acid Biosynthetic Process”, “Positive Regulation of Acute Inflammatory Response”, and “Positive Regulation of Fatty Acid Metabolic Process” in response to THz stress response.

## 4. Discussion

This review systematically summarized an updated literature review of the effects of THz on gene expression profiling studies in different groups.

Only 13 studies met our inclusion criteria. Our knowledge of how terahertz-based wave irradiation can affect live tissues at a cellular level is scarce. Interest in THz technologies continues to grow but fewer studies have investigated the biological effects of terahertz-based wave irradiation on gene expression. As of now, no reviews summarizing the present evidence to draw more acute conclusions have been conducted on studies where changes at the transcript (mRNA) level were assessed as the outcome.

### 4.1. Risk of Bias Assessment

The case–control study was the only study design used to explore the link between THz and gene expression changes, and therefore, no element of the study design seemed to be the origin of the various results. Thus, it showed a low risk of bias. At least two replicates were used in each study, and they all specified the number of replicates, thereby representing a low risk of bias. As for the quality of exposure, all studies reported the exposure duration, with some missing details in the other parameters, indicating a high risk of bias. With regard to temperature, changes were reportedly monitored in all studies, while one study, Cheon et al. [34], did not report temperature change, meaning a low risk of bias. RNA extraction time post-exposure was reported in all but two studies: Bock et al. [10] and Zhao et al. [40]. Overall, the assessment of the risk of bias revealed that four studies provided all the parameters, while the remaining nine studies had at least one parameter missing.

### 4.2. Summary of Evidence

Although pathway analyses were performed for most studies, their different pathway nomenclature made comparisons throughout studies that are not comparable one to another. For completeness and to ensure fair comparisons, the mainly reported differentially expressed genes and enriched pathways are presented by topic in terms of the main findings on cellular types.

The skin was the most used model [8,22,32,34,37,38]. It is likely that more people will have to be subjected to electromagnetic THz waves in the near future, as their use has been envisioned to be fast-growing and will likely find wider applications in categories such as patients undergoing THz diagnostics, the security and the military fields, material science, art conservation workers, and communication networks. This raises concerns about dermal tissue that is continuously radiated with THz bandwidth and the adverse ocular effects. The skin is the principal recipient organ of terahertz applied to the body [41]. The depth and width penetration of THz radiation into the skin is estimated to be 0.1 to 0.3 mm and depends on the frequency of the wavelength. Hence, probable hazards may involve the skin’s superficial cellular layers [4]. Moreover, the skin is considered a good model since it plays the role of buffer between the external and internal environment; therefore, the electromagnetic energy is highly absorbed by the skin [18]. However, terahertz absorption by the skin is a complex process. In this perspective, Titova et al. [32] tested THz effects in dermatologic conditions and inflammatory skin diseases by radiating artificial human skin tissue. The keratinization formation of the cornified envelope genes was downregulated. Their findings set grounds for the possible use of high-THz pulses for therapeutic applications by targeting expression patterns of inflammatory-regulating genes, as they found a low expression of immunologic proteins (SERPINS). In the same line of work, Hough et al. [37,38] did a series of experiments on 3D skin tissue exposed to prolonged trains of THz. Both studies showed that terahertz pulses dysregulated pathways involved in human cancer initiation, maintenance, and progression in skin cells. More recently, Cheon et al. [34], in a 48 h irradiation period, assessed DNA methylation at 1.6 THz. They stimulated the global magnitude of DNA methylation by 16% in melanoma cancerous samples. The authors found a significantly decreased mRNA expression of the JUN/FOS transcription factor and inflammation-related gene (CXCL8). The findings also revealed that THz modulates transcriptional changes with potential applications in cancer, since abnormalities in transcriptional activities are known to initiate cancer. The result is of broader health importance due to the methylation that occurred in irradiated cells. Kim et al. [8] proposed a mechanism by which THz activates wound response in skin but the initial signaling trigger remains unclear. Xu et al. [22] explored the role THz might play in driving the repair of spinal injury. THz stimulation across control and treated groups elucidated molecular mechanisms promoting the rehabilitation of SCI (Spinal Cord Injury) after 28 days of daily THz irradiation treatment for 15 min.

Concerning immune cells, Echchgadda [36] investigated changes that take place immediately in jurkat cells exposed to THz compared to thermally heated cells. The results indicated that the response induced by THz could not be perfectly mimicked by heated cells. These studies warrant further investigations to support new insights into the potential applicability of THz in clinical settings.

Concerning corneal cells, Zhao et al. [39] found that the expression of numerous genes, known as protooncogenes, which are involved in normal cell growth and anaplastic lymphoma kinase (ALK), was enhanced, and human centromere-associated protein E (CENPE) expression was the only under-expressed gene in human corneal epithelial cells.

Out of the 13 studies, 3 studies [10,16,35] covered THz effects on stem cells. Stem cells are unspecialized cells capable of infinite renewal. They are able to intrinsically differentiate into any specialized cell of an organism. Developmental potency is diminished in each step, which means perturbations of the hallmarks of stem cells may cause developmental disorders and tissue depletion [42].

Bock et al. [10] was the earliest study to explore gene expression using microarrays and the first shred of evidence of THz radiation on stem cells. These studies explored to what extent THz radiation affects the differentiation capabilities of mouse MSCs. Both investigations [10,16] resulted in expression profile changes in genes involved in differentiation. This brings about skeptical questions of whether THz radiation modifies self-renewal ability and the narrow differentiation capabilities of stem cells. They found increased expression transcriptional regulator activity induced by THz radiation through promoting the differentiation of stem cells into adipocytes. This early research indicated the non-thermal biological effects of THz and theorized that the variation in specific genes could be explained by the DNA breathing model, which put forward the claim that the responses of genes to THz radiation are prone to the promoter structure, therefore enabling transcription as a result of denaturation bubbles upon THz exposure [43]. Furthermore, it was hypothesized that THz radiation could be a biological modifier tool for the differentiation of specialized cells.

Concerning neuronal cells, two studies were found. Zhao et al. [40] showed that Cytotoxicity effects were not observed in response to THz exposure. A downstream analysis of the pathways results appeared to be in line with the morphological changes. Shang et al. [31] reported an increased expression of Aqp5 and Mmp3; meanwhile, they observed downregulation in Itpr1, Atp2b4, Apof, and Erf in neonatal rat neuron cells. The transcriptional activity of AP-1 was significantly diminished to binding sites, implying a possible direct modification of the interaction between DNA and TFs.

Peltek et al. [33] conducted the only study on bacteria. They showed that THz pulses induced a nonthermal response in gene expression. Electromagnetism leads to a stress response mediated by “pili adhesion”, “cell aggregation”, and “assembly of the septal ring”. The study revealed one characteristic of THz radiation for the targeted activation or repression of genes in bacteria.

Altogether, using the advantages of having datasets from GEO, we obtained, overall, 24 samples. A total of 415 differentially expressed genes were screened and put together. Of which there were 21 from GSE41083, 98 from GSE41084, only 4 from GSE41085, 259 from GSE44671, and finally 33 from GSE178729. The meta-analysis focused on consistent changes in every single dataset. All unique differentially expressed genes were included in the study. This method was able to capture both shared and individual signals that would otherwise be missed in single datasets. The meta-analysis demonstrated that there is a high heterogeneity change during exposure. The heterogeneity may arise from various factors, including dose dependency, differences in types of biological materials being tested, and variations in the parameters of the terahertz wave. Nevertheless, there was no prominent consistent change in gene expression linked to heat stress. Considering all of this together, we conclude that several frequencies and power densities are capable of promoting non-thermal changes in the gene expression of different cell types and in regulating pathways.

### 4.3. Exposure Parameters

Two main factors are to be considered when assessing the biological effects of electromagnetic THz radiation; one is the irradiation parameters and the second is the nature of the biological material. Although there are some consistent and highly mixed patterns of effects shown by the authors, the diversity in exposure parameters and biological models made it hard to identify and single out one or several biomarkers. In light of this, it can be said that the degree of THz absorption and the biological response to the stress with regard to THz exposure depends on various factors, including the frequency, power density, and exposure time, as well as the biological system properties and cell types, which implies the heterogeneity in differential responses. Moreover, the biological impacts could be attributed to modifications in the cell molecular structure in THz-exposed samples [44,45].

These observations raise two problems of great importance in the field of terahertz exposure: the reproducibility and replicability of the results and the impact of the biological model. The lack of uniformity among protocols renders increasingly difficult challenges to replicating studies individually. The findings of terahertz experiments are difficult to explain and compare because of the variety of models, frequencies, exposure systems, power densities, and exposure durations. Additionally, attention is often drawn to the variations in frequencies and exposure conditions to compare differences between THz studies, and as a consequence, the influence of the biological material is, in many instances, minimized.

Moreover, it is hypothesized that exposure in terms of incident power density on a culture medium has a limited impact on the cells, which may have been far away from where the THz energy is absorbed. Or, in the case where it reaches the cells, it might not be ubiquitously distributed. Thus, better methods of irradiation techniques are necessary for high-quality THz biological effects studies.

### 4.4. Gene Expression Technology

Microarray and recently RNA-Seq have become powerful tools to solve a variety of biological problems in study designs such as exposed versus unexposed groups, with the biological goal of detecting possible statistically differential expression [46,47].

RNA-seq appears to perform better in gene expression profiling compared to microarray. Log_2_ fold change (FC) and false discovery rate (FDR) are often used to screen differentially expressed genes (DEGs). DEGs results tables consist of all genes that have an FDR of less than 0.05 as hard thresholds and are statistically associated with a particular condition. It is noteworthy that a decrease in FC results in an increase in the number of DEGs [48]. Differences in thresholding applied to fold change will give different sets of DEGs as a consequence of different processes, signaling pathways, and functions involved as a result of biological experiments [49]. The assumption is that genes that react the most have the highest fold change; therefore, expression changes by a factor of 1.5 or 2 are much more interesting than genes that do not really change. The above methods have drawbacks. Trivial phenotypical changes can be caused by a trivial variation in expression. Variability in FC is beneficial in reducing or increasing gene expression data, the power of analysis, and background noise. Fold change increase leads towards a decrease in the number of DEGs. Genes can be significantly expressed with low fold changes. The mathematical and biological meaning resulting from global differential gene expression is not always the same. It is good to identify DEGs but it is also crucial to put it into a biological framework. The biological context of fold change is likely linked to both the genes and the experimental context [50].

In addition, pathways analyses are derived only from about 1% of the original data collected versus 99% discarded with their biological meanings. An increasing number of researchers apply gene ontology or pathway enrichment analysis to better fit the study purpose. Gene expression and transcriptomic technologies produce a tremendous amount of quantitative data, usually an extended list of transcripts that are overwhelming to comprehend. Roughly 150 to 600 genes are hard to decipher and overwhelming. In addition, some genes are promoters and transcription factors that control the activity of other interesting sets of genes. Genes tend to be team players in networks in order to exhibit a distinct biological function. A way to deal with this issue is the use of bioinformatics tools to establish and investigate which of the genes share a pathway or network and, therefore, work towards a unique function. Pathway analysis helps to derive meaning from these high-throughput experiments with different features, validate or confirm expected results, generate new hypotheses, and design subsequent experiments.

GO (Gene Ontology) [51], KEGG (Kyoto Encyclopedia for Genes and Genomes) [52], IPA (Ingenuity Pathway Analysis) [53], and Reactome [54] provide pathways analysis approaches such as over-representation analysis (ORA) and GSEA (Gene Set Enrichment Analysis). One problem encountered with ORA and GSEA is not only the major differences between these curated databases in their terminology but also the differences in the data underlying pathway definitions. Each pathway is defined by a set of genes that may not exactly be the same and render pathway descriptions slightly differently across databases. Terms may overlap across databases or be unique to one database. Whereas GO is more specialized towards cellular function, KEGG is oriented towards metabolism pathways, while Reactome has a lot more disease-related pathways than GO and KEGG.

Furthermore, in gene interactions, the promoter directions are not considered, thereby producing false results in certain conditions. Moreover, gene expression studies using an observational design between non-exposed and exposed groups only provide a brief picture or snapshot of the biological process at a given time.

Time points might not be optimal for capturing gene expression alterations in the biological model used. The limited range of time points in RNA collection produces a weaker response or may provide false-negative results [55]. Longer studies and follow-ups may permit a better dissection of the biological variations. While it is impossible to determine the extent of which the platform type may have influenced the results, lots of the variability reported in the studies might be a direct result of the usage of different gene expression assessment technologies and pipelines for biological quantification and the applied statistical methods.

### 4.5. Limitations

One of the strengths of this study resides in its wide eligibility criteria, providing considerable heterogeneity among the included studies and an exhaustive search of multiple databases. We employed PRISMA guidelines and searched multiple databases. As in most systematic reviews, our review is limited by the heterogeneity of the outcome definitions. The studies differed with regard to parameters that they have used. A second limitation of this review is that the heterogeneity of the studies did not allow a strong meta-analysis to offer a larger picture of the differentially expressed gene pathways due to differences in the study methodology. Despite these limitations, we were still able to synthesize the data by listing the reported gene expression patterns. An additional limitation was excluding studies published not in English.

## 5. Conclusions

To conclude, this systematic review is the first that has investigated terahertz radiation studies using microarray or RNA-Seq. We undertook a review to assess the degree to which studies that had focused on gene expression led to overall conclusions. The review also highlighted the methodological variations across studies. A standardization of the methods for reporting results could enhance our understanding of THz biological effects and indicate which family genes are the most likely to exert their influence on THz stress response. There are several limitations of the research that need to be improved in coming studies, among which are better-designed longitudinal studies, mainly with larger samples and more than one exploratory method for more accurate observations. Our investigation consolidates and advances our understanding of the gene expression underlying THz exposure.

Finally, in the future, additional and more comprehensive studies should focus on the epigenetic underpinnings of the range of responses or evidence. Moreover, current breakthroughs in the field of bioinformatics such as metabolomics profiling and array-based methylation profiling could fill the voids and provide a more complete picture of the complex connection between THz and gene expression.

## Figures and Tables

**Table 2:** Gene expression profile approach and study outcomes.

Study	Gene Expression Profile Method	Log 2 Fold Change	Numbers of DEGs	Pathways	Biological Outcomes
Bock et al. [10]	Microarray	NA	2204	NA	Gene related to the following: Adiponectin ↑; GLUT 4 ↑; FABP4 ↑; PPARG ↑; PPARA ↓; LXR ↓; Cyclin B ↓; TBP ↓
Alexandrov et al. [16]	Microarray	0	20	GO	Gene related to the following: Adiponectin ↑; FABP4 ↑; PPARG ↑; Nfe2l2 ↓; Gem-; Slco4a1 ↓ Gem ↓; Slco4a1 ↓; Arg2 ↓; Ifitm6 ↓; Hspa1a ↓; Hspa1b ↓; Hipk1 ↓; Slc6a12 ↓; 4921509J17Rik ↓; Hspa4l ↓; Rhpn2 ↓; Akr1b3 ↓; Flrt3 ↓; Tet2+; Ctgf ↑; Skil ↓; Fgd3 ↓; Nfe2l2 ↑; Dclre1b ↓; Fn1↓
Kim et al. [8]	Microarray	1.5	149	IPA	Gene related to the following: inflammation (TGF- β ↑); wound healing response (Bmp2 ↑, Cd44 ↑, Thbs1 ↑, Serpine1 ↑, Krt6a ↑, Lep ↑, Sprrb1 ↑)
Tivota et al. [32]	Microarray	0.6	442		Gene related to the following: psoriasis (S100 family: S100A11 ↓, S100A15 ↓, S100A12 ↓; Small Proline Rich Region: SPRR1B ↓, SPRR2A ↓, SPRR3 ↓, SPRR2B ↓, SPRR2C ↓, LCE3D ↓, involucrin (IVL) ↓; Serine protease inhibitor: SerpinB3 ↓, SerpinB4 ↓, SerpinB7 ↓, SerpinB13 ↓); atopic dermatitis ↓; epidermal hyperplasia ↓; dermatitis ↓; cancer (DEFB103A ↓, DEFB4 ↓, DEFB1 ↓, LOC728454 ↓, CD24 ↓); apoptotic signaling pathways (S100A ↓, S100P ↓)
Echchgadda et al. [36]	Microarray	2	531	IPA	Gene related to the following: cytoplasm (ARSB ↓, CYP19A1 ↓, CYP2D6+, CYP2E1 ↓, CYP4X1 ↑, GOT1L1 ↑, MAOB ↑, PCYT1B ↑, PLD1 ↑, TAT ↑, ATP2A1 ↑, BHMT ↑, CAD ↓, CTPS2 ↑, SARDH ↑, ACTA1 ↑, ACTA2 ↑, MYH4 ↓, RHOB ↑, RHOJ ↑, WASL ↑, STON2 ↑, MAP2K5 ↑, PDE1A ↑, PIK3C2A ↑, PLCB4 ↓, PLD1 ↑, RAB11B ↑, RAB7A ↑, RGS7 ↓, SOS2 ↓); plasma membrane (CD44 ↓, ATP2B3 ↑, CDH19 ↑, DSP ↑, ITGB1 ↑, ITGB3 ↓, ADRA1D ↑, DRD1 ↓, FGFR3 ↑, GABBR2 ↑, GNA11 ↑, NPR3 ↑, NTRK2 ↑, PTGER3 ↑,); cell shape, adhesion and cytoskeleton organization; immune response (HLA-DPA1 ↓, HLA-DQA1 ↑, IGHG1 ↑, IL4 ↑); transcription (ATF2 ↑, APBB1 ↑, JUN ↑, TNL2 ↑, RGS12 ↑); extracellular space (CANT1 ↓, FGF10 ↓, FGF7 ↓, PLAU ↑, ALB ↓, APOE ↑)
Echchgadda et al. [36]	Microarray	2	531	IPA	Gene related to the following: cytoplasm (ARSB ↓, CYP19A1 ↓, CYP2D6+, CYP2E1 ↓, CYP4X1 ↑, GOT1L1 ↑, MAOB ↑, PCYT1B ↑, PLD1 ↑, TAT ↑, ATP2A1 ↑, BHMT ↑, CAD ↓, CTPS2 ↑, SARDH ↑, ACTA1 ↑, ACTA2 ↑, MYH4 ↓, RHOB ↑, RHOJ ↑, WASL ↑, STON2 ↑, MAP2K5 ↑, PDE1A ↑, PIK3C2A ↑, PLCB4 ↓, PLD1 ↑, RAB11B ↑, RAB7A ↑, RGS7 ↓, SOS2 ↓); plasma membrane (CD44 ↓, ATP2B3 ↑, CDH19 ↑, DSP ↑, ITGB1 ↑, ITGB3 ↓, ADRA1D ↑, DRD1 ↓, FGFR3 ↑, GABBR2 ↑, GNA11 ↑, NPR3 ↑, NTRK2 ↑, PTGER3 ↑,); cell shape, adhesion and cytoskeleton organization; immune response (HLA-DPA1 ↓, HLA-DQA1 ↑, IGHG1 ↑, IL4 ↑); transcription (ATF2 ↑, APBB1 ↑, JUN ↑, TNL2 ↑, RGS12 ↑); extracellular space (CANT1 ↓, FGF10 ↓, FGF7 ↓, PLAU ↑, ALB ↓, APOE ↑)
Bogomazova et al. [35]	Microarray	1.5	73	GO	Gene related to the following: mitochondrial ribosome (MRPL34 ↑, MRPL43 ↑, MRPL55 ↑, MRPS24 ↑)
Hough et al. [37,38]	Microarray	1.5	1681	GO and KEGG	Gene related to the following: Ras–Raf–Mek–Erk Cascade (KRAS ↓, RAS ↓, MAPK3 ↓); apoptosis (AKT ↑); pro-inflammatory AND cytokine–cytokine receptor interaction (CXCL16 ↓, BMP7 ↓, CCL8 ↑, CXCL5 ↑, CCL20 ↑, Il-6 ↑, IL-24 ↑); glioma pathway (CXCL16 ↓, BMP7 ↓, CCL8 ↑, CXCL5 ↑, CCL20 ↑, Il-6 ↑, IL-24 ↑, HGF ↑, RAP1B ↑, FGF2 ↑, FGF7 ↑, RAC2 ↓, RRAS ↓, EFNA1 ↓, PGF ↓, calmodulin family: CALML5 ↓, CALML3 ↓, CALML1 ↓, CAMK families ↓), calmodulin (CaM ↓), calmodulin-like (CaLM ↓), and calmodulin-dependent kinase (CaMK) families, GNAI3 ↓, EDNRB ↑, PPP3CC ↑.
Zhao et al. [39]	RNA-Seq	1.5	HCE^+^ Müller^+^ RPE = 766	GO	Gene related to the following: cell growth, differentiation and migration (RET ↑, ALK ↑, ROS1 ↑); fibroblast growth factor (FGFR2 ↑); stable spindle microtubule (CENPE ↓); MUC16 ↓; COL1A2 ↑
Peltek et al. [33]	RNA-Seq	2 (up) 1 (down)	741	KEGG	Gene related to the following: cell aggregation (*tdcABCDEFGR*, and *matA-F* genes); suppression of cell motility (gene *yjjQ*); suppression of cell division (*dicABCF*, *FtsZ*, and *minCDE*); adhesin synthesis (*sfmACDHF*); cell envelope stabilization (*yjbEFGH*, *gfcA*)|pilus ↑; molecular functions of fimbrial porins ↑; organization and assembli of pili ↑; cell adhesion ↑; cytoplasmic genes ↓; genes of the respiratory chain ↓; ribosomes ↓; processes of aerobic respiration ↓; translation ↓
Shang et al. [31]	RNA-Seq	1	165	GO and KEGG	Gene related to the following: ribosome (small subunit: Rps18, Rps28; large subunit: Rp136, Rp136A, Rp135; mitoribosomal: Mrps18c, Mrps15, Mrps11; Mrp114; Mrp118, Mrp158, Mrp121) ↑; calcium signaling pathway; axon guidance; insulin signaling pathway; GTPase MF: Rab GTPase binding; guanyl-nucleotide exchange factor activity; GTPase binding; small GTPase binding; Ras GTPase binding Aquaporin (Aqp5) ↑; matrix metallopeptidase (Mmp3) ↑; transcriptional factor (Erf) ↓; alipoprotein (Apof) ↓; plasma membrane calcium ATPase (Atp2b4) ↓; intracellular receptor for inositol (Itpr1)↓
Zhao et al. [40]	Microarray	1.5	1249	GO and KEGG	Gene related to the following: neuron projection ↑; synapse organization ↑; dendritic spine ↑; actin binding ↓; cytoskeleton organization ↓; response to stimulus ↓ Ppp1r2 ↑, Camk2b ↑, Fmr1 ↑, Nlgn1 ↑, Nrxn1 ↑, Rapgef ↑, Brwd3 ↓, Crx ↓, Olfr18 ↓, Svil ↓, Tas2r1 ↓, Tnf ↓
Cheon et al. [34]	Microarray	2		KEGG	Gene related to the following: cancer, apoptosis pathways, and inflammation related-gene (transcription factor for AP-1: *JUN*, *FOS*, *CXCL8* ↓)
Xu et al. [22]	RNA-Seq	1	137	GO and KEGG	Gene related to the following: inflammation; immune regulation; IL-17 signaling pathway (alipoprotein C-I ↑; alipoprotein C-IV ↑; immunoglobulin lambda constant 1 ↑; immunoglobulin heavy constant γ 2B ↑)

Notes. ↑ upregulation; ↓ downregulation.

**Table 3:** Dataset collected and included in the meta-analysis.

Dataset	Author	Year	GSE ID	Platform	Cell Type	Total Sample
Dataset_1	Alexandrov et al. [16]	2013	GSE41083	GPL1261 [Mouse430_2] Affymetrix	Mouse stem cells	6
Dataset_2	Alexandrov et al. [16]	2013	GSE41084	GPL1261 [Mouse430_2] Affymetrix	Mouse stem cells	6
Dataset_3	Alexandrov et al. [16]	2013	GSE41085	GPL1261 [Mouse430_2] Affymetrix	Mouse stem cells	6
Dataset_4	Kim et al. [8]	2013	GSE44671	GPL6246 [MoGene-1_0-st] Affymetrix	C57BL/6J and BALB/c skin cell	6
Dataset_5	Zhao et al. [40]	2021	GSE178729	GPL21163 Array Agilent-074809	Mouse cortical neurons and oligodendrocytes precursor cells	6

## Data Availability

No new data were created. Data sharing is not applicable to this article.

## Supplementary Materials

The following supporting information can be downloaded at: https://www.mdpi.com/article/10.3390/genes15081045/s1, Table S1. Gene set enrichment analysis (C5 Gene Ontology) of differentially expressed genes (p.adjust < 0.05).
