# Supplementary material for: Non-Thermal Effects of Terahertz Radiation on Gene Expression: Systematic Review and Meta-Analysis
Source: Genes (Basel). 2024 Aug 8;15(8):1045. doi: 10.3390/genes15081045 (PMC11354197; doi:10.3390/genes15081045)
Supplement: Supplementary file 1 [file genes-15-01045-s001.zip › genes-3143633-supplementary.pdf]

Effects of Terahertz Radiation on Gene Expression: A Systematic Review

Mactar Ndiaga Dione[1] , Sen Shang[1] , , Zhang Qi [1], Zhao Sicheng [1], Xiaoyun Lu[1,2,£]

[1]Xi'an Jiaotong University (XJTU), School of Life Science, Xi'an, China

[2] Key Laboratory of Biomedical Information Engineering of Ministry of Education, School of Life Science and Technology, Xi'an Jiaotong University, Xi'an 710049, Shaanxi, P. R. China

[£]Last author

Search strategy for the systematic review of gene expression following THz exposure using databases

a) PubMed

Search string:

1: "Terahertz (THz)" OR "terahertz radiation*" OR "terahertz wave*" OR "THz" OR "terahertz exposure" OR "terahertz pulse*"

2: "terahertz radiation"[MeSH Terms]

3: Terahertz Radiation/adverse effects[MAJR]

4: #1 OR #2 OR #3

5: "DNA methylation*" OR "DNA damage" OR "Epigenetic modification" OR "gene network" OR "chromosomal aberration" OR "transcription factor*" OR transcription* OR "transcriptional change*" OR "transcriptional activity"

6: "Oligonucleotide Array Sequence Analysis"[MeSH Terms] OR "Sequence Analysis, RNA"[MeSH Terms] OR "RNA-seq*" OR "mRNA" OR "Gene Expression"[MeSH Terms] OR "gene expression" OR "gene expression regulation" OR "gene expression profiling" OR "Microarray Analysis"[MeSH Terms] OR "transcriptome profiling" OR "transcriptom*" OR "Gene Ontology"[MeSH Terms]

7: "metabolomic* analysis" OR "metabonomic*" OR "metabolite*"

8: #5 OR #6 OR #7

9: #4 AND #8

64 hits on 28/02/2024

b) Web of Science

Search string: TS=(("terahertz radiation*" or "terahertz wave*" or "THz" or "THz puls*") and ("biological effect*" or "bioeffect*" or "gene expression" or "expression of genes" or "gene expression profiling" or "gene expression regulation" or "RNA sequencing" or "rna sequencing" or "microarray analysis" or "epigenetic modification" or "gene network" or "transcripton*" or "transcriptomic

analysis" or "gene ontology" or "transcriptional changes" or "genetic transcription" or "transcription factor" or "transcriptional activity" or "gene network" or "transcriptome profiling" or "metabo*" or "metabonomic" or "transcriptomics" or "metabonome" or "metabolic" or "metabolite*" or "metabolomic analysis"))

191 hits on 28/02/2024

c) Scopus

Search string: TITLE-ABS ("Terahertz radiation*" OR "THz radiation" OR "terahertz pulse*" OR "terahertz wave*") AND TITLE-ABS ("Gene Expression" OR "gene network" OR "genetic transcription" OR "epigenetic modification" OR "RNA-sequencing" OR "Expression Gene*" OR "gene expression profiling" OR "Gene Expression Profiling" OR "Gene expression profiles" OR "gene ontology" OR "transcriptom*" OR "transcriptomic analysis" OR "transcriptomics" OR "transcriptome profiling" OR "Transcription, Genetic" OR "Reverse Transcription" OR "Transcription Elongation, Genetic" OR "Transcriptome" OR "dna methylation" OR "metabol*")

66 hits on 28/02/2024

d) Embase

Search string: ('terahertz radiation*' OR 'terahertz radiation*':ti,ab,kw OR 'terahertz pulse*' OR 'terahertz pulse*':ti,ab,kw OR 'terahertz wave*' OR 'terahertz wave*':ti,ab,kw OR 'thz radiation'/exp OR 'thz radiation' OR 'thz radiation':ti,ab,kw) AND ('biological activity'/exp OR 'biological activity' OR 'biological activity':ti,ab,kw OR 'biological effect'/exp OR 'biological effect' OR 'biological effect*':ti,ab,kw OR 'genotoxicity'/exp OR 'genotoxicity' OR 'genotoxicity':ti,ab,kw OR 'gene expression regulation'/exp OR 'gene expression regulation' OR 'gene expression regulation':ti,ab,kw OR 'gene expression'/exp OR 'gene expression' OR 'gene expression':ti,ab,kw OR 'gene expression profiling'/exp OR 'gene expression profiling' OR 'gene expression profiling':ti,ab,kw OR 'epigenetic modification':ti,ab,kw OR 'epigenetic modification'/exp OR 'epigenetic modification' OR 'gene network'/exp OR 'gene network' OR 'gene network':ti,ab,kw OR 'rna sequencing'/exp OR 'rna

sequencing' OR 'rna sequencing':ti,ab,kw OR 'gene ontology'/exp OR 'gene ontology' OR'gene ontology':ti,ab,kw OR 'high throughput sequencing'/exp OR 'high throughput sequencing' OR 'high throughput sequencing':ti,ab,kw OR 'genetic transcription'/exp OR 'genetic transcription' OR 'genetic transcription':ti,ab,kw OR 'transcriptomic analysis'/exp OR 'transcriptomic analysis' OR 'transcriptomic analysis':ti,ab,kw OR 'transcriptome'/exp OR 'transcriptome' OR 'transcriptome':ti,ab,kw OR 'transcriptome profiling'/exp OR 'transcriptome profiling' OR 'transcriptome profiling':ti,ab,kw OR 'dna methylation'/exp OR 'dna methylation' OR 'dna methylation':ti,ab,kw OR 'demethylation'/exp OR 'demethylation' OR 'demethylation':ti,ab,kw OR 'transcription initiation'/exp OR 'transcription initiation' OR 'transcription initiation':ti,ab,kw OR 'metabolomics'/exp OR 'metabolomics' OR 'metabolomics':ti,ab,kw OR 'metabolome'/exp OR 'metabolome' OR 'metabolome':ti,ab,kw OR 'metabonomic'/exp OR 'metabonomic' OR 'metabonomic':ti,ab,kw OR 'metabonome':ti,ab,kw)

61 hits on 28/02/2024

e) ProQuest

Search string:

S1: noft("Terahertz" OR "terahertz radiation*" OR "terahertz wave*" OR "THz" OR "terahertz exposure" OR "terahertz pulse*")

S2: Terahertz adverse effects

S3: [S1] OR [S2]

S4: noft("DNA methylation*" OR "DNA damage" OR "Epigenetic modification" OR "gene network" OR "chromosomal aberration" OR "transcription factor*" OR transcription* OR "transcriptional change*" OR "transcriptional activity")

S5: noft("Sequence Analysis" OR "RNA-seq*" OR "mRNA" OR "Gene Expression" OR "gene expression" OR "gene expression regulation" OR "gene expression profiling" OR "Microarray Analysis" OR "transcriptome profiling" OR "transcriptom*" OR "Gene Ontology")

S6: noft("metabolomic analysis" OR "metabonomic*" OR "metabolite*")

S7: [S4] OR [S5] OR [6]

S8: [S3] AND [S8]

243 hits on 28/02/2024

Eligibility criteria for the systematic review

Articles were included only if it was a:

- peer-reviewed study addressing gene expression in terahertz radiation;
- Frequency range from 0 to 10 THz
- In vitro and animal models studies

Articles were excluded when it was a study that:

- had not been carried out on humans, such as animal studies, *in vitro* and *in silico* studies;
- articles with only abstracts available;
- were categorized as conference proceedings, letters, comments, reviews, or editorials;
- had no appropriate study design, i.e. not being considered as a case-control;
- duplicate articles;
- articles that quantitatively analyzed gene expression levels via qPCR;
- had no English full text available

Supplementary Table S1. Gene set enrichment analysis (C5 Gene Ontology) of differentially expressed genes (p.adjust < 0.05)

Gene Set Name	Gene Set Name
GOCC_INTERMEDIATE_FILAMENT	GOCC_INTERMEDIATE_FILAMENT_CYTOSKELETON
GOBP_POSITIVE_REGULATION_OF_FATTY_ACID_METABOLIC_PROCESS	HP_PALMAR_HYPERHIDROSIS
GOMF_SECONDARY_ACTIVE_TRANSMEMBRANE_TRANSPORTER_ACTIVITY	GOCC_KERATIN_FILAMENT
GOBP_POSITIVE_REGULATION_OF_FATTY_ACID_BIOSYNTHETIC_PROCESS	HP_PLANTAR_HYPERKERATOSIS
GOMF_HEPARIN_BINDING	GOBP_POSITIVE_REGULATION_OF_ACUTE_INFLAMMATORY_RESPONSE
GOBP_ACUTE_PHASE_RESPONSE	HP_ALOPECIA
GOBP_BICARBONATE_TRANSPORT	HP_NAIL_DYSTROPHY
HP_ABNORMALITY_OF_THE_PLANTAR_SKIN_OF_FOOT	GOBP_ACUTE_INFLAMMATORY_RESPONSE
GOMF_OXALATE_TRANSMEMBRANE_TRANSPORTER_ACTIVITY	HP_ABNORMALITY_OF_THE_NAIL
HP_HYPERKERATOSIS	GOBP_REGULATION_OF_ACUTE_INFLAMMATORY_RESPONSE
GOBP_POSITIVE_REGULATION_OF_INTERLEUKIN_8_PRODUCTION	GOBP_OXALATE_TRANSPORT
GOBP_BROWN_FAT_CELL_DIFFERENTIATION	GOMF_GLYCOSAMINOGLYCAN_BINDING
GOBP_POSITIVE_REGULATION_OF_MAPK_CASCADE	GOMF_INTEGRIN_BINDING
GOMF_BICARBONATE_TRANSMEMBRANE_TRANSPORTER_ACTIVITY	GOBP_POSITIVE_REGULATION_OF_DEFENSE_RESPONSE
GOBP_POSITIVE_REGULATION_OF_INFLAMMATORY_RESPONSE	GOMF_ACTIVE_ION_TRANSMEMBRANE_TRANSPORTER_ACTIVITY
HP_THICKENED_SKIN	GOBP_REGULATION_OF_UNSATURATED_FATTY_ACID_BIOSYNTHETIC_PROCESS
GOMF_ORGANIC_ANION_TRANSMEMBRANE_TRANSPORTER_ACTIVITY	GOCC_APICAL_PLASMA_MEMBRANE
GOCC_BASAL_PART_OF_CELL	GOMF_SECONDARY_ACTIVE_SULFATE_TRANSMEMBRANE_TRANSPORTER_ACTIVIT Y
HP_PALMOPLANTAR_HYPERHIDROSIS	GOBP_SODIUM_ION_TRANSPORT
GOBP_SKIN_DEVELOPMENT	GOBP_GRANULOCYTE_CHEMOTAXIS

Supplementary Table S1. Continued

GOBP_EPITHELIAL_CELL_PROLIFERATION	GOMF_ACTIVE_TRANSMEMBRANE_TRANSPORTER_ACTIVITY
GOBP_REGULATION_OF_FATTY_ACID_BIOSYNTHETIC_PROCESS	GOBP_LEUKOCYTE_CHEMOTAXIS
GOCC_BASOLATERAL_PLASMA_MEMBRANE	GOBP_FAT_CELL_DIFFERENTIATION
GOBP_POSITIVE_REGULATION_OF_NEUROINFLAMMATORY_RESPONSE	HP_PARONYCHIA
GOBP_CELLULAR_KETONE_METABOLIC_PROCESS	GOBP_POSITIVE_REGULATION_OF_LIPID_BIOSYNTHETIC_PROCESS
GOBP_CYTOKINE_MEDIATED_SIGNALING_PATHWAY	GOBP_POSITIVE_REGULATION_OF_CYTOKINE_PRODUCTION
GOBP_ANTERIOR_POSTERIOR_PATTERN_SPECIFICATION	GOBP_CELL_CHEMOTAXIS
GOBP_INTERLEUKIN_8_PRODUCTION	GOBP_REGULATION_OF_FATTY_ACID_METABOLIC_PROCESS
GOBP_KERATINOCYTE_PROLIFERATION	GOMF_DICARBOXYLIC_ACID_TRANSMEMBRANE_TRANSPORTER_ACTIVITY
GOMF_CHLORIDE_TRANSMEMBRANE_TRANSPORTER_ACTIVITY	GOMF_SULFATE_TRANSMEMBRANE_TRANSPORTER_ACTIVITY
GOCC_CELL_CELL_JUNCTION	GOBP_SULFATE_TRANSPORT
GOMF_ORGANIC_ACID_TRANSMEMBRANE_TRANSPORTER_ACTIVITY	GOMF_ANION_TRANSMEMBRANE_TRANSPORTER_ACTIVITY
GOBP_POSITIVE_REGULATION_OF_RESPONSE_TO_EXTERNAL_STIMULUS	GOBP_POSITIVE_REGULATION_OF_PEPTIDYL_TYROSINE_PHOSPHORYLATION
GOBP_PEPTIDE_CROSS_LINKING	HP_ABNORMALITY_OF_THE_PERIUNGUAL_REGION
HP_PATCHY_ALOPECIA	GOBP_GRANULOCYTE_MIGRATION
GOBP_REGULATION_OF_INFLAMMATORY_RESPONSE	GOBP_NEUTROPHIL_CHEMOTAXIS
GOBP_REGULATION_OF_KERATINOCYTE_PROLIFERATION	GOBP_SODIUM_ION_TRANSMEMBRANE_TRANSPORT
GOCC_APICAL_PART_OF_CELL	GOBP_RESPONSE_TO_TEMPERATURE_STIMULUS
GOBP_ORGANIC_ANION_TRANSPORT	GOMF_SOLUTE_ANION_ANTIPORTER_ACTIVITY
